# Gender-Specific Associations of CYP4F2 (rs1558139) Gene Polymorphism and Serum Levels in Early and Exudative Age-Related Macular Degeneration

**DOI:** 10.7759/cureus.91988

**Published:** 2025-09-10

**Authors:** Alvita Vilkeviciute, Rasa Ciumbaraite, Enrika Pileckaite, Loresa Kriauciuniene, Ho Hin Ma, Raimonda Piskiniene, Rasa Liutkeviciene

**Affiliations:** 1 Laboratory of Ophthalmology, Neuroscience Institute, Medical Academy, Lithuanian University of Health Sciences, Kaunas, LTU; 2 Faculty of Medicine, Lithuanian University of Health Sciences, Kaunas, LTU; 3 Department of Ophthalmology, Medical Academy, Lithuanian University of Health Sciences, Kaunas, LTU

**Keywords:** age-related macular degeneration, cyp4f2 serum, early amd, exudative amd, gene polymorphism, rs1558139

## Abstract

Background: The study aimed to assess the potential association between *CYP4F2* rs1558139 gene polymorphism, serum CYP4F2 levels, and the development of age-related macular degeneration (AMD).

Materials and methods: A total of 204 patients with early AMD, 204 patients with wet AMD, and 294 healthy individuals as controls participated in this study. Genotyping of rs1558139 was carried out using real-time polymerase chain reaction (RT-PCR), while levels of serum CYP4F2 protein were determined by enzyme-linked immunosorbent assay (ELISA).

Results: The allele A at *CYP4F2 *rs1558139 was statistically significantly less frequent in females with early AMD than in the control females (46.8% vs. 54.77%, p=0.037). However, in the exudative AMD group, the AA genotype was more frequent in women than in men (31.9% vs. 17.3%, p<0.001). Serum CYP4F2 concentration was higher in early and exudative AMD groups than in controls (5.394 (3.554) vs. 1.599 (4.142), p=0.013 and 8.422 (8.356) vs. 1.599 (4.142), p<0.001, respectively).

Conclusions: The *CYP4F2 *rs1558139 polymorphism shows gender-specific associations with AMD. The A allele is less frequent only in females with early AMD, while the AA genotype is more common in women with exudative AMD. A higher serum CYP4F2 level may contribute to the development of AMD.

## Introduction

Age-related macular degeneration (AMD) is a neurodegenerative disease that affects more than 2 million people in Europe. It is a multifactorial pathological condition characterized by the progressive degeneration of photoreceptor cells and retinal pigment epithelial cells in the macular of the retina, resulting in an irreversible central vision loss at the beginning and eventually blindness [1]. Our retina structure changes because of aging. Consequently, drusen accumulate beneath the retinal pigment epithelium (RPE), resulting in changes in the appearance of Bruch’s membrane (BrM) [2].

AMD is a multifactorial pathological disease influenced by various factors, including aging, psychological stress, oxidative stress, inflammatory processes, changes in the extracellular matrix, biologically active alterations in the retinal pigment epithelium (RPE), and genetic predisposition, all of which interact in complex ways [3]. In addition, epidemiological studies suggest that women may have a slightly higher prevalence of AMD compared to men, particularly for bilateral forms of the disease [2]. Identification of multiple genetic loci suggests several critical biological pathways in AMD pathogenesis such as complement pathway, extracellular/collagen matrix pathway, oxidative stress pathway, angiogenesis signaling pathway, and cholesterol and lipid metabolism pathways [4-6]. Furthermore, several recently performed studies on AMD pathogenesis have focused on the genes involved in lipid metabolism [4,6].

Approximately 40% of the volume of drusen consists of lipids [7]. Therefore, lipid metabolism, particularly high-density lipoprotein (HDL), is believed to be involved in the development of AMD. Lipid particles accumulate within BrM before early AMD development. Furthermore, a strong genetic component has been shown to be associated with a high risk of AMD development [8]. Genetic variants in lipid metabolism genes have also been found, resulting in the deposition of lipids and the formation of drusen beneath the RPE and BrM, leading to the manifestation of AMD [9].

In the pathogenesis of AMD, inflammation and oxidative stress play key roles in the development of AMD [10]. During inflammation, the enzyme CYP450, which is a derived oxylipin [11], has elevated levels due to an increase in the expression of CYP4F2, CYP4A, and CYP4F3 family enzymes.

Enzymes of the CYP4 family play a key role in the metabolism of pro-inflammatory arachidonic acid derivatives, such as 20-hydroxyeicosatetraenoic acid (20-HETE) and leukotriene B4, thereby contributing to the regulation of inflammatory responses. Notably, 20-HETE has been shown to impair endothelial insulin signaling and enhance vascular endothelial growth factor (VEGF) activity through the activation of NADPH oxidase [12]. VEGF promotes angiogenesis and cell proliferation [13], increases vascular permeability and vasodilation, and elevates protease activity within the retina. These processes facilitate vascular remodeling and the formation of new blood vessels, ultimately contributing to the development of choroidal neovascularization [14]. Given the established role of lipid metabolism and inflammation in AMD pathogenesis, and prior associations of *CYP4F2* variants with related vascular and metabolic diseases, we employed a hypothesis-driven approach to investigate whether the *CYP4F2* rs1558139 polymorphism is associated with AMD risk. Accordingly, this study aimed to assess the potential association between the *CYP4F2* rs1558139 gene polymorphism, serum CYP4F2 levels, and AMD development.

## Materials and methods

The study was approved by the Ethics Committee for Biomedical Research (No. BE-2-/13), and all participants provided written informed consent in accordance with the Declaration of Helsinki.

Study population

The study population comprised three groups: patients with early AMD (n=204), exudative AMD (n=204), and healthy individuals as a control group (n=294). The control group was formed by considering the distribution of patients in both early and exudative AMD groups based on age and sex (Table 1). The early AMD group consisted of 68.63% women (n=140) and 31.37% men (n=64), while the wet AMD group included 66.18% women (n=125) and 33.82% men (n=69). In the control group, 72.45% were women (n=213) and 27.55% were men (n=81). Participant ages ranged from 50 to 90 years in the early AMD group (median: 66), 50 to 93 years in the wet AMD group (median: 69), and 50 to 90 years in the control group (median: 68). There were no significant differences in age or gender distribution among the groups (p>0.05). Considering the other parameters, we found that LDL-Chol was significantly elevated in both the early and exudative AMD groups compared to the controls (p<0.001) (Table 1).

**Table 1:** Demographic characteristics of the study population. TChol: total cholesterol; LDL-C: low-density lipoprotein cholesterol; HDL-C: high-density lipoprotein cholesterol. ^*^TChol, LDL-C, HDL-C, and TG were evaluated in 150 persons (50 persons in each group). The p-value indicates the level of significance, with statistically significant differences observed when p<0.05.

Characteristic	Group			p-value
	Early AMD, n=204	Exudative AMD, n=204	Control, n=294	
Gender, n (%) Men/women	64 (31.37)/140 (68.63)	69 (33.82)/135 (66.18)	81 (27.55) / 213 (72.45)	0.302
Age, median (range)	66 (50–90)	69 (50–93)	68 (50–90)	0.127
Smoking history, n (%) Never/current or former	180 (88.2)/24 (11.8)	190 (93.1)/14 (6.9)	272 (92.5)/22 (7.5)	0.145
TChol, mean (SD), mmol/L*	7.08 (1.48)	7.18 (1.26)	6.47 (1.36)	0.092
LDL-C, mean (SD), mmol/L*	7.00 (1.45)	7.25 (1.24)	4.72 (2.76)	<0.001
HDL-C, mean (SD), mmol/L*	1.41 (0.29)	1.49 (0.41)	1.44 (0.29)	0.562
TG, mean (SD), mmol/L*	1.71 (1.21)	1.75 (0.91)	1.71 (1.21)	0.349

The study included patients diagnosed with early or exudative AMD based on AAO criteria and OCT findings. A control group comprised ophthalmologically healthy individuals scheduled for cataract surgery, with no ocular or systemic comorbidities. Blood samples were collected immediately prior to surgery to avoid potential confounding effects related to surgical intervention. All participants underwent a comprehensive ophthalmologic evaluation, and inclusion/exclusion criteria were strictly applied to eliminate confounding systemic or ocular conditions. Ophthalmological evaluation, DNA extraction, and genotyping were widely described and mentioned in our previous studies [15,16].

Selection of *CYP4F2* rs1558139 for analysis

The CYP4F2 rs1558139 polymorphism was selected based on a hypothesis-driven approach. CYP4F2 encodes a cytochrome P450 enzyme involved in lipid metabolism and inflammatory regulation, both of which are critical processes in the pathogenesis of AMD. Previous studies have reported associations between CYP4F2 variants, including rs1558139, and diseases characterized by vascular dysfunction and lipid imbalance, such as hypertension and coronary artery disease [17,18]. Given the overlapping mechanisms, we hypothesized that rs1558139 may also contribute to AMD susceptibility. Therefore, we focused our analysis on this SNP to explore its potential role in AMD.

Determination of CYP4F2 protein levels

Serum CYP4F2 concentrations in AMD patients were measured using a commercially available human CYP4F2 ELISA kit (Abcam, Cambridge, United Kingdom), following the manufacturer’s protocol. Optical density was recorded immediately at a wavelength of 450 nm using a microplate reader (Multiskan FC, Thermo Scientific, Waltham, MA, United States). CYP4F2 levels were quantified based on a standard curve, with a detection range of 0.63-40 ng/mL and a sensitivity of 132 pg/mL. 

Statistical analysis

Statistical analyses were conducted using IBM SPSS Statistics for Windows, Version 27.0 (Released 2019; IBM Corp., Armonk, New York, United States). The Shapiro-Wilk test was applied to evaluate the normality of continuous variables such as age and serum protein levels. Results were expressed as median and interquartile range (IQR), and comparisons between groups were made using the Mann-Whitney U test for non-normally distributed data. Categorical variables (gender, genotype, allele distributions) are shown as counts (percentages) and compared using the chi-square test. Hardy-Weinberg equilibrium for CYP4F2 rs1558139 was tested in controls using χ².

The association between selected SNPs and the risk of early and exudative AMD was assessed using binomial logistic regression. Results were expressed as odds ratios (ORs) with corresponding 95% confidence intervals (CIs). Multiple genetic inheritance models were tested, including codominant (comparison of heterozygotes and minor allele homozygotes each against major allele homozygotes); dominant (grouping minor allele homozygotes and heterozygotes versus major allele homozygotes); recessive (comparing minor allele homozygotes against the combined group of heterozygotes and major allele homozygotes); overdominant (heterozygotes versus both types of homozygotes), and additive (trend across genotypes: major allele homozygotes, heterozygotes, and minor allele homozygotes). Model fit was evaluated using the Akaike information criterion (AIC), with the model showing the lowest AIC value considered the most appropriate. A p-value below 0.05 was considered indicative of statistical significance.

## Results

*CYP4F2* rs1558139 analysis in early and exudative AMD

The genotype and allele frequencies of rs1558139 in the control group were consistent with Hardy-Weinberg equilibrium expectations (Table 2). Comparative analysis showed no statistically significant differences in the distribution of rs1558139 genotypes between individuals with early or exudative AMD and the control group (p>0.05) (Table 2). When applying binomial logistic regression analysis in the patients with early and exudative AMD and the control group, no significant differences were shown between the patients with early AMD and the control subjects (Table 3). 

**Table 2:** Frequency of CYP4F2 rs1558139 genotype in early and exudative AMD and control groups. AMD: age-related macular degeneration; HWE: Hardy-Weinberg equilibrium. The p-value indicates the level of significance, with statistically significant differences observed when p<0.05. *Early AMD vs. control group. **Exudative AMD vs. control group.

Gene marker	Genotype/allele	Control group (n=294), n (%)	p-value HWE	Early AMD group (n=204), n (%)	p-value*	Exudative AMD group (n=204), n (%)	p-value**
*CYP4F2* rs1558139	GG	89 (30.3)	0.182	59 (28.9)	0.334	68 (33.3)	0.723
	GA	135 (45.9)		87 (42.6)		106 (52.0)	
	AA	70 (23.8)		49 (24.0)		49 (24.0)	
	G	313 (53.2)		185 (45.3)	0.657	185 (45.3)	0.603
	A	275 (46.8)		224 (54.9)		224 (54.9)	

**Table 3:** Binomial logistic regression analysis in patients with early and exudative AMD. AMD: age-related macular degeneration; OR: odds ratio; CI: confidence interval; AIC: Akaike information criteria. The p-value indicates the level of significance, with statistically significant differences observed when p<0.05.

Model	Genotype	OR (95% CI)	p-value	AIC
Patients with early AMD				
Codominant	GA	1.184 (0.781;1.795)	0.425	677.814
	AA	0.840 (0.504;1.402)	0.505	
Dominant	GA+AA	1.067 (0.721;1.579)	0.746	677.914
Recessive	AA	1.322 (0.851;2.053)	0.214	676.452
Overdominant	GA	1.274 (0.891;1.822)	0.185	676.259
Additive	A	0.937 (0.729;1.203)	0.608	677.756
Patients with exudative AMD				
Codominant	GA	0.843 (0.557;1.277)	0.421	679.371
	AA	0.916 (0.565;1.485)	0.722	
Dominant	GA+AA	0.868 (0.592;1.273)	0.470	677.498
Recessive	AA	1.012 (0.666;1.537)	0.957	678.017
Overdominant	GA	0.876 (0.611;1.255)	0.470	677.498
Additive	A	0.949 (0.746;1.208)	0.673	677.841

*CYP4F2* rs1558139 analysis by age and gender

When comparing the rs1558139 genotype and allele frequency between men and women in the early AMD group, it showed that the A allele was statistically significantly less frequent in women with early AMD than the female control group (p=0.037). However, in the exudative AMD group, the AA genotype was more frequent in women than in men (p<0.001) (Table 4).

**Table 4:** Frequency of CYP4F2 rs1558139 genotype in patients with early and exudative AMD and control subjects by gender. AMD: age-related macular degeneration. The p-value indicates the level of significance, with statistically significant differences observed when p<0.05. ^*^p<0.001.

Genotype/allele	Men		p-value	Women		p-value
	AMD group, n (%)	Control group, n (%)		AMD group, n (%)	Control group, n (%)	
Patients with early AMD						
GG	21 (32.8)	31 (38.3)	0.601	38 (27.2)	58 (27.2)	1.0
GA	33 (51.6)	36 (44.4)	0.083	73 (52.1)	99 (46.5)	0.328
AA	10 (15.6)	14 (17.3)	0.826	29 (20.7)	56 (26.3)	0.254
G	75 (58.9)	98 (60.5)	0.744	149 (53.2)	199 (45.23)	0.037
A	53 (41.4)	64 (39.5)		131 (46.8)	241 (54.77)	
Patients with exudative AMD						
GG	27 (38.3)	31 (38.3)	1	41 (30.4)	58 (27.2)	0.544
GA	36 (44.4)	36 (44.4)	0.413	51 (37.8)	99 (46.5)	0.121
AA	6 (17.3)*	14 (17.3)	0.155	43 (31.9)*	56 (26.3)	0.274
G	90 (65.22)	98 (60.5)	0.399	133 (49.26)	199 (45.23)	0.296
A	48 (34.78)	64 (39.5)		137 (50.74)	241 (54.77)	

When the rs1558139 genotype frequency was compared by age and gender, no significant differences were able to be revealed either in patients with early or wet AMD (data not shown). Binomial logistic regression analysis was performed in patients with early and exudative AMD, as well as in control group subjects, by gender and age. The AA genotype was more common in women with exudative AMD aged ≥65 years than in men with early AMD aged <65 years (31.2% vs. 8.0%, p=0.0228) (Tables 5, 6). The GA genotype under the overdominant model showed a 2.8-fold greater risk of early AMD in the male group aged <65 years (p=0.043) (Table 7). Meanwhile, when analyzing younger males and females with exudative AMD by age, no statistically significant differences were found (data not shown).

**Table 5:** Frequency of CYP4F2 rs1558139 genotypes in women and men with early AMD and control subjects by age. AMD: age-related macular degeneration. The p-value indicates the level of significance, with statistically significant differences observed when p<0.05. ^*^p=0.0228.

Genotype/allele	<65 years		p-value	≥65 years		p-value
	AMD group, n (%)	Control group, n (%)		AMD group, n (%)	Control group, n (%)	
Women						
GG	14 (25.9)	47 (27.8)	0.743	24 (27.9)	11 (25.0)	0.257
GA	29 (53.7)	81 (47.9)		44 (51.2)	18 (40.9)	
AA	11 (20.4)	41 (24.3)		18 (20.9)	15 (34.1)	
G	57 (52.8)	175 (51.8)	0.943	92 (53.5)	40 (45.5)	0.274
A	51 (47.2)	163 (48.2)		80 (46.5)	48 (54.5)	
Men						
GG	7 (28.0)	20 (40.8)	0.103	14 (35.9)	11 (34.4)	0.603
GA	16 (64.0)	19 (38.8)		17 (43.6)	17 (53.1)	
AA	2 (8.0)*	10 (20.4)		8 (20.5)	4 (12.5)	
G	30 (60.0)	59 (60.2)	1.000	45 (57.7)	39 (60.9)	0.826
A	20 (40.0)	39 (39.8)		33 (42.3)	25 (39.1)	

**Table 6:** Frequency of CYP4F2 rs1558139 genotypes in women and men with exudative AMD and control subjects by age. AMD: age-related macular degeneration. The p-value indicates the level of significance, with statistically significant differences observed when p<0.05. ^*^p=0.0228.

Genotype/allele	<65 years		p-value	≥65 years		p-value
	AMD group, n (%)	Control group, n (%)		AMD group, n (%)	Control group, n (%)	
Women						
GG	7 (26.9)	47 (27.8)	0.501	34 (31.2)	11 (25.0)	0.749
GA	10 (38.5)	81 (47.9)		41 (37.6)	18 (40.9)	
AA	9 (34.6)	41 (24.3)		34 (31.2)*	15 (34.1)	
G	24 (46.15)	175 (51.78)	0.545	109 (50.0)	40 (45.45)	0.553
A	28 (53.85)	163 (48.22)		109 (50.0)	48 (54.55)	
Men						
GG	8 (61.5)	20 (40.8)	0.352	19 (33.9)	11 (34.4)	0.855
GA	4 (30.8)	19 (38.8)		32 (57.1)	17 (53.1)	
AA	1 (7.7)	10 (20.4)		5 (8.9)	4 (12.5)	
G	20 (76.92)	59 (60.2)	0.178	70 (62.5)	39 (60.94)	0.965
A	6 (23.08)	39 (39.8)		42 (37.5)	25 (39.06)	

**Table 7:** Binomial logistic regression analysis in patients with early AMD and controls, according to their age and gender. AMD: age-related macular degeneration; OR: odds ratio; CI: confidence interval; AIC: Akaike information criteria. The p-value indicates the level of significance, with statistically significant differences observed when p<0.05.

Model	Genotype	OR (95% CI)	p-value	AIC
Women				
<65				
Codominant	GA	1.202 (0.578;2.499)	0.622	252.284
	AA	0.901 (0.368;2.202)	0.819	
Dominant	GA+AA	01.101 (0.549;2.206)	0.787	250.809
Recessive	AA	1.252 (0.592;2.650)	0.557	250.529
Overdominant	GA	1.260 (0.682;2.329)	0.460	250.336
Additive	A	0.961 (0.624;1.479)	0.857	250.850
≥65				
Codominant	GA	1.120 (0.456;2.756)	0.805	169.751
	AA	0.550 (0.205;4.795)	0.236	
Dominant	GA+AA	0.861 (0.376;1.973)	0.724	170.277
Recessive	AA	1.954 (0.868;4.399)	0.106	167.812
Overdominant	GA	1.513 (0.726;3.155)	0.269	169.170
Additive	A	0.734 (0.441;1.220)	0.232	168.960
Men				
<65				
Codominant	GA	2.406 (0.811;7.140)	0.114	95.979
	AA	0.571 (0.100;3.273)	0.530	
Dominant	GA+AA	0.947 (0.625;5.030)	0.281	97.460
Recessive	AA	2.949 (0.593;14.653)	0.186	96.588
Overdominant	GA	2.807 (1.034;7.619)	0.043	94.939
Additive	A	0.981 (0.505;2.012)	0.981	98.659
≥65				
Codominant	GA	0.786 (0.279;2.216)	0.649	102.707
	AA	1.571 (0.374;6.611)	0.538	
Dominant	GA+AA	0.935 (0.351;2.492)	0.894	101.718
Recessive	AA	0.554 (0.150;2.040)	0.374	100.915
Overdominant	GA	0.682 (0.266;1.745)	0.424	101.095
Additive	A	1.143 (0.584;2.237)	0.697	101.584

Serum CYP4F2 concentration

**Figure 1:**
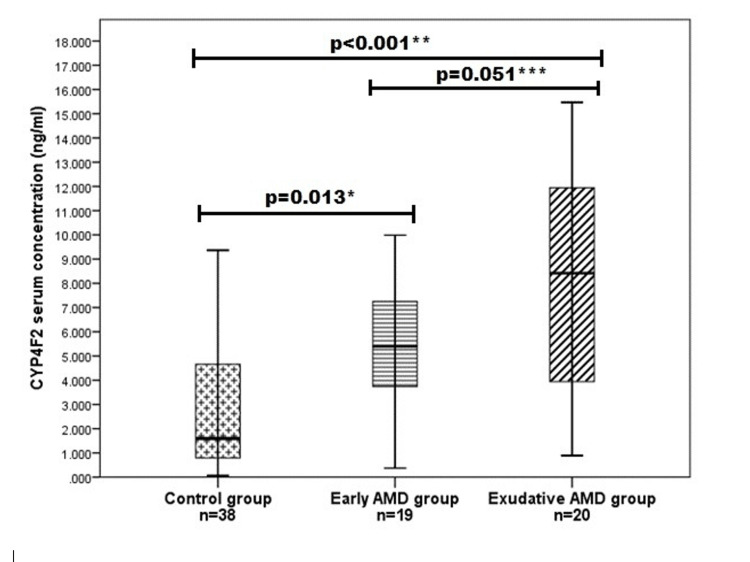
Serum CYP4F2 concentrations in the study groups. AMD: age-related macular degeneration. The bars represent the median with interquartile range (25th-75th quartile).

CYP4F2 ELISA test was performed on 80 samples: early AMD (n=20), exudative AMD (n=20), and controls (n=40). One sample from early AMD and two from the control group did not reach the minimum value and were all excluded from the statistical analysis. According to our data, we found that serum CYP4F2 concentration was higher in early and exudative AMD groups than in controls (median (IQR): 5.394 (3.554) vs. 1.599 (4.142), p=0.013 and 8.422 (8.356) vs. 1.599 (4.142), p<0.001, respectively). However, no differences were found between early and exudative AMD groups (median (IQR): 5.394 (3.554) vs. 8.422 (8.356), p=0.051) (Figure 1). CYP4F2 concentration was analyzed across CYP4F2 rs1558139 genotypes (GG, GA, and AA) in the overall sample and within each study group separately; however, no statistically significant differences were observed.

## Discussion

Our study investigating the association between the *CYP4F2* rs1558139 polymorphism and serum CYP4F2 levels with AMD adds novel insights to the complex genetic and biochemical landscape of AMD. To better position our findings, we compare them with previous ophthalmic and AMD-related genetic research.

Lipid metabolism and AMD

Our findings revealed significantly elevated LDL-cholesterol (LDL-C) levels in both early and exudative AMD groups compared to controls (p<0.001). This is consistent with previous evidence linking lipid metabolism to AMD pathogenesis, where LDL-C contributes to drusen formation through lipid accumulation beneath the retinal pigment epithelium (RPE) and Bruch’s membrane [19], oxidized LDL induces oxidative stress and inflammation [20], and elevated lipid levels, including total cholesterol, are associated with increased circulating angiogenic cytokines such as VEGF-A, VEGF-C, VEGF-D, and PlGF [21].

Nevertheless, conflicting results have also been reported concerning the associations between serum HDL concentrations, AMD, and genes involved in lipid metabolism. While some studies support a positive association [8,22], others found no significant links [23-27] or reported contradictory outcomes. Numerous investigations into systemic lipoproteins and lipid-related genes have likewise yielded inconsistent results [25-33]. These inconsistencies highlight the complex and multifactorial nature of lipid dysregulation in AMD.

*CYP4F2* rs1558139 polymorphism and AMD

This study primarily investigated the association of the *CYP4F2* rs1558139 polymorphism with early and exudative AMD. To the best of our knowledge, this is the first study to explore this specific SNP in these AMD subtypes. We observed that the rs1558139 A allele was statistically significantly less frequent in women with early AMD than in control women (46.8% vs. 54.77%, p=0.037), while it was more frequently observed in women with exudative AMD compared to men (31.9% vs. 17.3%, p<0.001). Additionally, the A/A genotype was more common in women with exudative AMD aged ≥65 years than in men with early AMD aged <65 years (31.2% vs. 8.0%, p=0.0228).

Only one previous study has analyzed *CYP4F2* rs1558139 in AMD patients, specifically in the atrophic AMD subtype, and reported no significant associations. However, that study included a small cohort [34]. In contrast, other polymorphisms of the same gene have been investigated. Our previous work on CYP4F2 rs2108622 demonstrated significant associations with central retinal thickness (CRT), with CC genotype carriers exhibiting increased CRT compared to CT and TT carriers [16].

Gender-specific associations

Our data suggest a gender-specific association of the *CYP4F2* rs1558139 polymorphism with AMD. The differential frequency of the A allele and A/A genotype between male and female patients indicates potential interactions between genetic variants and sex-specific biological mechanisms. Hormonal status, immune responses, and sex-specific metabolic regulation may influence how genetic polymorphisms modulate disease susceptibility and progression.

Gender-specific effects have also been reported in genetic studies of cardiovascular and metabolic diseases. For instance, age and sex interactions have been noted for several *CYP4F2* variants, particularly in relation to blood pressure regulation, lipid metabolism, and cardiovascular risk [35-39]. Russo et al. demonstrated age- and gender-dependent associations for vascular-related genotypes [40], and Edelman et al. observed genotype-gender interactions that influence disease expression [41]. In the context of *CYP4F2*, some polymorphisms such as rs2108622 have shown stronger associations in men, particularly in studies on stroke and hypertension in Asian populations [42-45].

Emerging evidence suggests that some AMD-associated SNPs may have sex-specific effects as well, although this aspect of AMD genetics remains underexplored. The *CFH* Y402H variant (rs1061170), one of the most extensively studied, has shown a stronger association with AMD in males in certain cohorts, potentially reflecting sex-based differences in immune regulation or complement system activation [46,47]. In contrast, the *ARMS2* A69S variant (rs10490924) has been reported to confer a slightly higher risk in females in some studies, though the effect size differences are modest and inconsistent across populations [48]. Additionally, previous studies have reported that the *CFH* I62V variant, along with risk alleles in both *ARMS2* and *CFH* I62V, are more frequently found in women than in men, potentially contributing to the higher observed risk of early AMD in women [48-50]. Other authors identified a potential gender-specific genetic association between the *MMP2* rs2287074 polymorphism with AMD in older women. The protective A allele was less frequent among women with AMD, particularly in those with late-stage disease, suggesting a possible role in disease progression [51].Together, these findings highlight the potential relevance of sex in modulating genetic susceptibility to AMD and underscore the importance of sex-stratified analyses in future studies.

Moreover, our findings point to a stronger involvement of CYP4F2 polymorphisms in women with AMD, which differs from previously reported male-dominant effects in cardiovascular traits. This discrepancy suggests that the functional impact of CYP4F2 may be disease- and tissue-specific and influenced by sex-based regulatory mechanisms. The observed elevation of serum CYP4F2 levels, particularly in women, supports this hypothesis. Future investigations are warranted to explore how sex-specific expression, hormonal regulation, or epigenetic mechanisms might influence CYP4F2 function in the retina. 

Comparison with major AMD loci

Recent large-scale meta-analyses have identified up to 63 AMD-associated genetic loci, including nine novel loci not reported in earlier GWAS [52]. In total, over 50 independent loci have been linked to AMD susceptibility, highlighting its polygenic nature and implicating key biological pathways such as complement activation, lipid metabolism, extracellular matrix remodeling, angiogenesis, and mitochondrial function.

The most consistently replicated and high-impact genes include *CFH*, *ARMS2*/*HTRA1*, and *APOE*. The *CFH* Y402H (rs1061170) variant impairs complement regulation, increasing inflammation and AMD risk [53,54]. *ARMS2* A69S and the *HTRA1* promoter variant (rs11200638), both located on chromosome 10q26, have been linked to mitochondrial dysfunction and extracellular matrix changes in the retina [55,56]. The *APOE *gene shows isoform-specific effects: the ε2 allele increases risk, while the ε4 allele appears protective, likely due to differences in cholesterol transport and immune modulation [57]. Together, these loci explain a substantial portion of AMD heritability -CFH variants alone may account for 30-50% of cases [58]. In contrast, variants like CYP4F2 rs1558139 likely have more modest effects and may act synergistically with these major risk genes or through secondary pathways.

Serum CYP4F2 levels in AMD

We also investigated serum CYP4F2 levels, which were significantly higher in both early and exudative AMD groups compared to controls (5.394 [3.554] vs. 1.599 [4.142], p=0.013; and 8.422 [8.356] vs. 1.599 [4.142], p<0.001, respectively). Notably, no prior studies have examined this association in AMD. One study on angina pectoris found that CYP4F2 enzyme levels were significantly lower in patients compared to controls, suggesting context-dependent regulation [59]. In contrast, Tatarunas et al. found that elevated CYP4F2 protein levels, particularly in CYP4F2*13 carriers and clopidogrel users, may contribute to increased platelet reactivity [60].

Although CYP4F2 is involved in the biosynthesis of 20-HETE, we did not observe a direct correlation between CYP4F2 and 20-HETE serum levels in our AMD patients. This suggests that additional regulatory mechanisms, including tissue-specific expression and post-translational modifications, may modulate 20-HETE production independently of circulating CYP4F2. The increased CYP4F2 expression in AMD patients, particularly in women and those with exudative disease, may reflect a compensatory response to oxidative stress, inflammation, or vascular dysfunction, rather than a strictly pathogenic or protective mechanism.

Limitations and future directions

A limitation of our study is that we evaluated only a single SNP (rs1558139) in the *CYP4F2* gene and did not perform broader genotyping or genome-wide association screening. Although this variant was selected based on biological plausibility and prior evidence linking CYP4F2 to lipid metabolism and inflammatory pathways, other variants within CYP4F2 or interacting loci may contribute to AMD risk.

Moreover, our results suggest that CYP4F2 may influence AMD through both genetic and non-genetic mechanisms, including gene expression regulation, environmental factors, or disease-induced epigenetic changes. These findings emphasize the importance of future studies that incorporate comprehensive genotyping approaches, functional characterization of CYP4F2 in retinal tissue, investigation of sex-specific gene expression, and larger, multiethnic patient cohorts.

## Conclusions

Our study demonstrates a significantly higher prevalence of the A/A genotype of *CYP4F2* rs1558139 in women compared to men with exudative AMD, suggesting a possible sex-specific genetic influence on disease susceptibility. The presence of the A allele at rs1558139 may confer a protective effect against the development of early AMD in females. Furthermore, serum CYP4F2 concentrations were elevated in both early and exudative AMD groups compared to controls, indicating that alterations in CYP4F2 expression may be associated with AMD pathophysiology. These findings highlight the potential value of CYP4F2 as a biomarker and underscore the importance of considering sex differences in AMD genetic studies.
